# Gamma-Ray and Thermal Neutron Shielding of Fe-Based Bilayer Composites with a Boron-Enriched Matrix and Tungsten Surface Coatings: Lead Benchmarks Included

**DOI:** 10.3390/ma18225208

**Published:** 2025-11-17

**Authors:** Jiho Chai, Ku Kang, Ho Sub Chi, Changwoo Kang, Sangmin Lee, Jin Kook Kim

**Affiliations:** 1 Department of Defense Protective Engineering, Seoul National University of Science and Technology, Seoul 01811, Republic of Korea; chisoo2001@naver.com; 2 ROK Chemical, Biological and Radiological Defense Research Institute, Seoul 06790, Republic of Korea; bisu9082@gmail.com (K.K.); sosurim3@gmail.com (H.S.C.); 3 Strategic Studies, Graduate School of National Security, Korea National Defense University, Nonsan-si 33021, Republic of Korea; cwkang530@gmail.com; 4 Center for Security and Strategy, Korea Institute for Defense Analyses, Seoul 02455, Republic of Korea; smlee6453@gmail.com

**Keywords:** radiation shielding, gamma-ray attenuation, thermal neutrons, Fe-based composites, tungsten coatings, lead-free materials, aerospace applications

## Abstract

This study investigates the design and experimental evaluation of Fe–B–Si-based bilayer composites engineered for dual shielding against gamma rays and thermal neutrons. The materials integrate a boron-enriched amorphous Fe matrix with surface coatings of high-Z fillers—lead (Pb) and tungsten (W)—dispersed in an epoxy resin. W or Pb powders (20–40 µm) were dispersed in epoxy resin at a high filler loading (60–70 wt% metal, approximately several tens to one by weight). This ensured a dense and uniform coating structure. The metallic fillers were high-purity (≥99.9%) powders. Gamma-ray attenuation was examined using ^137^Cs and ^60^Co sources at photon energies of 661.7, 1173, and 1332 keV, while thermal neutron shielding was assessed with a moderated Am-Be neutron source. The effects of boron concentration (13–21 at%) in the matrix and coating thickness (80–400 μm) were systematically evaluated. Increasing boron content markedly enhanced thermal neutron attenuation, reaching up to 29%, whereas Pb- and W-filled coatings achieved more than 85% gamma-ray attenuation at 661.7 keV. All measurements were repeated three times; standard deviations were below 2% across conditions, confirming reproducibility and indirectly indicating uniform coating dispersion. At 661.7 keV, the half-value and tenth-value layers (HVL/TVL) were derived from the measured linear attenuation coefficients to benchmark performance. Notably, W coatings delivered shielding efficiency comparable to Pb while offering advantages in environmental safety, mechanical robustness, and regulatory compliance. These results highlight the potential of Fe–B–Si bilayer composites as lightweight, scalable, and lead-free shielding materials for aerospace electronics, portable radiation protection devices, and modular panels for satellites and nuclear facilities.

## 1. Introduction

Exposure to cosmic radiation has emerged as a growing concern with the expansion of aerospace and high-altitude aviation activities, especially along polar flight paths and orbital missions. Gamma rays and thermal neutrons generated from cosmic ray interactions in the upper atmosphere are among the primary radiation threats encountered in such environments, posing risks to electronic systems and biological tissue alike [1,2,3]. These challenges have underscored the need for effective radiation shielding materials that are not only efficient but also lightweight and structurally adaptable—especially for aerospace, defense, and other radiation-sensitive applications [4,5,6,7].

Conventional shielding materials present inherent limitations when faced with complex radiation fields. Aluminum (Al), commonly used in aerospace structures, exhibits poor gamma-ray attenuation due to its low atomic number and density [8,9]. Lead (Pb), while effective against gamma radiation, presents significant drawbacks including toxicity, high density, and weak neutron-shielding capability [10,11,12]. Furthermore, much of the existing literature has focused on shielding against a single radiation type, with relatively few studies addressing materials capable of attenuating both gamma rays and thermal neutrons in an integrated design [13,14,15]. This gap limits the development of multifunctional shielding systems suitable for space missions, compact electronics, and human-rated vehicles [16,17].

To address these limitations, this study proposes the use of Fe-based composite materials incorporating boron (B) and tungsten (W) for dual radiation shielding. Boron is a well-established neutron absorber due to its high thermal neutron capture cross-section, while tungsten offers high atomic number and density, making it effective for gamma-ray attenuation and a promising non-toxic substitute for lead [11,18,19,20,21,22]. Although Fe-based amorphous materials have previously been explored for electromagnetic and structural applications [23], their potential for combined gamma and neutron shielding remains underexplored—especially in composite systems utilizing variable B content and high-Z surface coatings [24,25,26].

Recent studies (2016–2024) on polymer–metal shielding systems report 80–90% attenuation at 662 keV for uniformly dispersed W- or Pb-filled epoxies, providing quantitative baselines for comparison [6,9,27]. By contrast, few works have paired such coatings with B-enriched Fe matrices to realize integrated γ/*n* protection. The present work closes this gap by explicitly correlating boron fraction and coating thickness with measured attenuation metrics, including HVL/TVL, to deliver reproducible, materials-focused insights.

The aim of this work is to experimentally assess the gamma-ray and thermal neutron shielding performance of Fe-based amorphous composites with tunable boron concentrations and Pb or W-filled epoxy coatings. Gamma-ray attenuation was measured at photon energies of 661.7, 1173, and 1332 keV, while thermal neutron shielding was evaluated using a moderated ^241^Am–Be neutron source. To facilitate reproduction, all key material and geometry parameters are specified in Section 2.1, Section 2.2, Section 2.3 and Section 2.4. This study demonstrates the feasibility of using bilayer Fe-based composites as scalable and lightweight alternatives to conventional shielding materials for use in aerospace, nuclear safety, and radiation-sensitive environments [28,29,30].

## 2. Materials and Methods

### 2.1. Composite Fabrication

**Figure 1:**
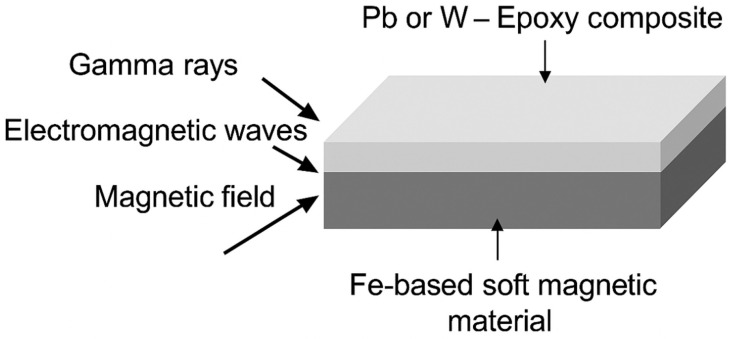
Schematic diagram of the Fe-based bilayer composite structure developed for dual shielding against gamma rays and thermal neutrons. The structure consists of a Pb- or W-filled epoxy surface layer and an underlying Fe–B–Si amorphous alloy substrate.

The Fe-based composite materials were fabricated in a bilayer architecture consisting of a soft magnetic substrate and a high-density surface coating. This configuration was designed to enable simultaneous attenuation of gamma radiation and thermal neutrons, while also considering the suppression of electromagnetic interference [10,11]. A schematic illustration of this bilayer structure is shown in Figure 1.

The base substrates were prepared using Fe–B–Si amorphous alloys, with systematic variation of the elemental composition to investigate the effect of boron content on radiation shielding behavior [23]. The resulting samples, designated as Group A, were produced with atomic ratios of Fe, B, and Si ranging from 70–78 at%, 13–21 at%, and fixed at 9 at%, respectively, as summarized in Table 1.

For the surface shielding layer, lead (Pb) or tungsten (W) powders were dispersed in a commercial-grade epoxy resin using mechanical stirring to ensure uniform particle distribution [11,21]. The resulting suspensions were applied onto the Fe-based substrates by casting, with coating thicknesses controlled at 80, 100, 150, 200, and 400 µm. The coated specimens were cured at room temperature for 24 hours to ensure complete polymerization and good interfacial adhesion between the layers. W or Pb powders (20–40 μm) were dispersed in epoxy resin at a high filler-to-resin ratio, typical for dense shielding composites. The powders were high-purity metals (≥99.9%), and no intentional oxide or compound additives were included in the formulation. The low-temperature cure minimized oxidation and residual stress, promoting stable adhesion.

### 2.2. Material Composition and Coating Structure

As outlined in the previous subsection, Group A samples consisted of Fe–B–Si amorphous alloys with systematically varied boron content. These uncoated specimens served as the reference group, allowing the evaluation of how the base matrix composition influences gamma-ray and thermal neutron attenuation.

Groups B and C were designed to assess the effect of high-Z surface coatings on shielding performance. Both groups employed the same substrate composition (Fe 78 at%, B 13 at%, Si 9 at%) as Sample #1 from Group A, ensuring that the coating layer was the sole variable. Group B utilized lead (Pb)-filled epoxy coatings, while Group C used tungsten (W)-filled epoxy. For both groups, coating thicknesses were systematically varied at five levels: 80, 100, 150, 200, and 400 µm. Although SEM/EDS imaging was not performed, the coating uniformity was inferred indirectly from statistical consistency: triplicate transmission measurements showed standard deviations below 2%, suggesting macroscopically homogeneous dispersion without significant agglomeration.

### 2.3. Experimental Setup for Gamma-Ray Shielding

**Figure 2:**
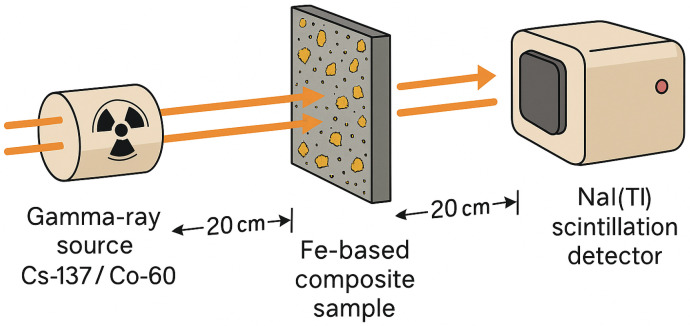
Schematic diagram of the experimental setup for gamma-ray and neutron shielding measurements.

The gamma-ray shielding performance of the composite samples was evaluated using a standard narrow-beam transmission setup. Two gamma-emitting radioisotopes were used: ^137^Cs (661.7 keV) and ^60^Co (1173 and 1332 keV). Each specimen was a circular disk (30 mm diameter, 10 mm total thickness). Source–sample and sample–detector distances were fixed at 20 cm, following recommended transmission geometry (e.g., ASTM D5168). A NaI(Tl) scintillation detector was positioned 20 cm downstream from the sample to record the transmitted gamma-ray intensity. All specimens were tested under identical geometric conditions to ensure reproducibility (Figure 2). All measurements were repeated three times; the resulting standard deviations (<2%) bound the combined experimental uncertainty for each condition.

The shielding efficiency (*η*) was calculated by comparing the measured gamma-ray intensities with and without the sample in place, using the following expression:

$$\eta \;\left(\%\right)=\left(1-\frac{I_{\mathrm{sample}}}{I_{\mathrm{open}}}\right)\times 100$$

where $I_{\mathrm{sample}}$ is the transmitted intensity with the sample, and $I_{\mathrm{open}}$ is the intensity recorded without any shielding. For benchmarking, linear attenuation coefficients μ were obtained from $T=I_{\mathrm{sample}}/I_{\mathrm{open}}=\exp\left(-\mu x\right)$, enabling derivation of HVL and TVL in Section 2.4.

### 2.4. Experimental Setup for Thermal Neutron Shielding

Thermal neutron shielding performance was evaluated using a ^241^Am–Be source, which emits fast neutrons in the energy range of 1–12 MeV. To obtain a thermalized neutron field, the emitted neutrons were moderated by a paraffin or high-density polyethylene block placed between the source and the sample. This moderation process reduced the neutron energy spectrum to near-thermal levels (∼0.025 eV), allowing evaluation of neutron attenuation as a function of boron content. The geometry (moderator placement and 20 cm source–sample–detector spacing) follows widely used Am–Be thermalization configurations, ensuring comparability with established datasets.

Each composite specimen was mounted vertically, and neutron transmission was measured using a BF_3_ proportional counter positioned 20 cm downstream of the sample, maintaining the same geometric configuration as in the gamma-ray experiments (Figure 2).

The thermal neutron shielding efficiency, $\eta_{n}$, was determined from the relative count rates with and without the sample in place:

(1) $$\eta_{n}\left(\%\right)=\left(1-\frac{C_{\mathrm{sample}}}{C_{\mathrm{open}}}\right)\times 100,$$

where $C_{\mathrm{sample}}$ is the neutron count rate measured behind the sample and $C_{\mathrm{open}}$ is the open-beam count rate recorded without shielding. All measurements were repeated three times, and the averaged values were used to reduce statistical uncertainty. Standard deviations were within 2% under all neutron conditions. As with γ tests, the low dispersion indirectly indicates uniform coating and stable interfaces.

We adopt $I=I_{0}e^{-\mu x}$ for photon attenuation; the Half-Value Layer (HVL) and Tenth-Value Layer (TVL) are defined as HVL=ln2/μ and TVL=ln10/μ, respectively. These metrics are used strictly for photon data and are reported from measured μ where applicable.

## 3. Results

### 3.1. Gamma-Ray Attenuation Performance

**Figure 3:**
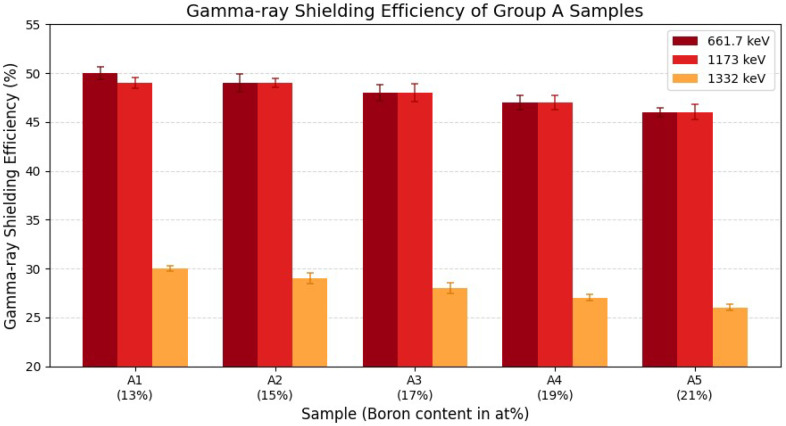
Gamma-ray shielding efficiencies of Fe–B–Si composite samples (Group A) measured at 661.7, 1173, and 1332 keV. Samples A1 to A5 have increasing boron contents from 13 at% to 21 at%. A gradual decrease in shielding efficiency is observed with increasing boron concentration, particularly at 661.7 keV. Error bars represent one standard deviation (1σ) of measurement uncertainty, confined within approximately 2% at maximum, indicating the reproducibility of the experimental measurements.

Figure 3 shows the gamma-ray shielding efficiencies of the Fe–B–Si composite samples (Group A), measured at photon energies of 661.7 keV (^137^Cs) and 1173 and 1332 keV (^60^Co). All samples had a uniform thickness of 10 mm, while the boron content was incrementally increased from 13 at% (A1) to 21 at% (A5), corresponding to a reduction in Fe content.

At 661.7 keV, the shielding efficiency decreased from 50% (A1) to 46% (A5). A similar trend was observed at 1173 keV, with efficiencies ranging from 49% to 46%. At the highest photon energy of 1332 keV, attenuation was lower overall, ranging from 30% to 26%. In all cases, a gradual reduction in gamma-ray shielding efficiency was observed with increasing boron content.

This reduction is attributed to the decreasing proportion of high-Z Fe atoms, which play a dominant role in gamma-ray attenuation via photoelectric and Compton scattering processes. The effect is more pronounced at lower photon energies, particularly 661.7 keV, where the photoelectric effect contributes significantly to attenuation.

### 3.2. Neutron Shielding Performance

**Figure 4:**
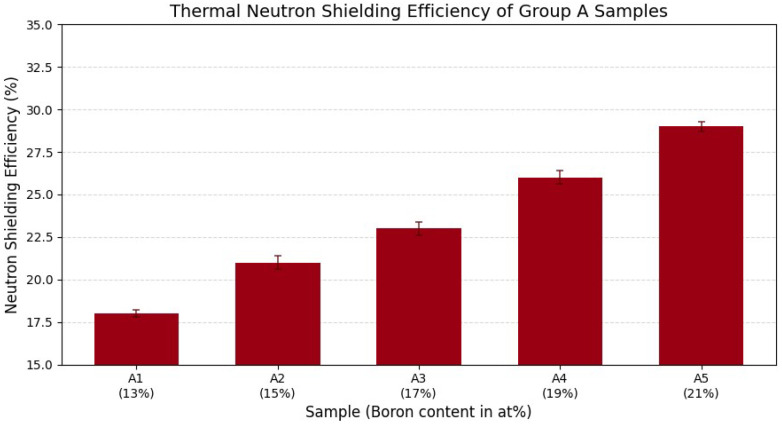
Thermal neutron shielding efficiencies of Fe–B–Si composite samples (Group A). Boron content increases from 13 at% (A1) to 21 at% (A5), resulting in a marked improvement in neutron attenuation, from 18% to 29%. Error bars represent one standard deviation (1σ) of measurement uncertainty, confined within approximately 2% at maximum, indicating the reproducibility of the experimental measurements.

Figure 4 presents the thermal neutron shielding efficiencies of Group A Fe–B–Si composite samples with boron contents ranging from 13 at% (A1) to 21 at% (A5). All samples had identical thickness and matrix structure, ensuring that only boron concentration varied.

The measured shielding efficiencies increased progressively with higher boron content: 18% (A1), 21% (A2), 23% (A3), 26% (A4), and 29% (A5). This consistent trend demonstrates that increasing B concentration enhances the composite’s thermal neutron attenuation capability, primarily due to the high neutron absorption cross-section of ^10^B through the ^10^B(n,α)^7^Li reaction.

These results confirm the effectiveness of boron incorporation in Fe-based composites for moderating thermal neutron exposure.

### 3.3. Gamma-Ray and Neutron Shielding Performance by Pb and W Coating Thickness

**Figure 5:**
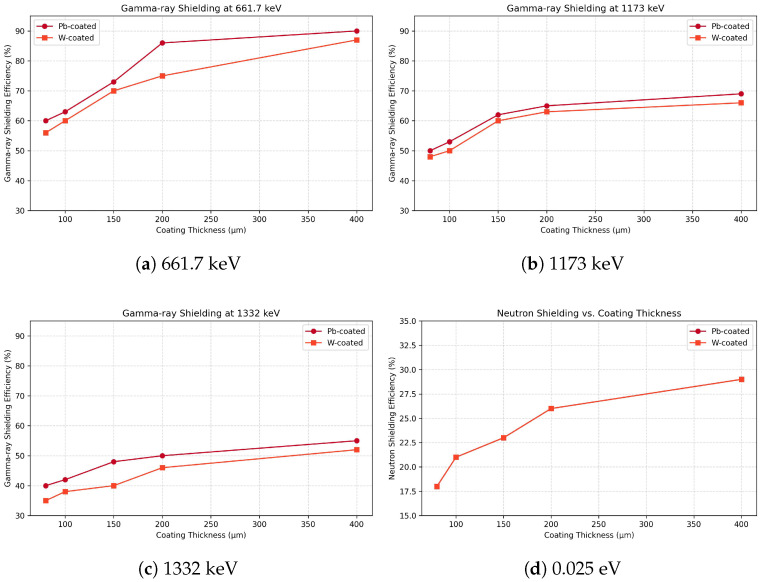
Shielding efficiencies of Pb- and W-coated Fe–B–Si composite samples as a function of surface coating thickness. (**a**–**c**): Gamma-ray attenuation at 661.7, 1173, and 1332 keV. (**d**): Thermal neutron shielding at 0.025 eV, showing identical trends for both coatings due to the boron-based absorption mechanism in the matrix. Error bars from repeated measurements are within ±2% and therefore not shown for clarity. Neutron results are reported as efficiency (%) only, with no conversion to $\Sigma_{R}$, HVL, or TVL.

Table 2 and Figure 5 summarize the gamma-ray and thermal neutron shielding efficiencies of Pb- and W-coated composite samples (Groups B and C) with varying surface coating thicknesses. All samples shared the same Fe–B–Si base composition as Sample A1 (Fe 78 at%, B 13 at%, Si 9 at%).

At 661.7 keV, gamma-ray shielding efficiency increased from 60% to 90% for Pb coatings and from 56% to 87% for W coatings as the thickness increased from 80 to 400 μm. Similar trends were observed at 1173 and 1332 keV, with Pb consistently showing slightly higher attenuation than W across all thickness levels, attributable to its marginally higher atomic number and density.

In contrast, thermal neutron shielding performance remained identical between Pb- and W-coated samples at each thickness level. Efficiency values increased from 18% (80 μm) to 29% (400 μm), a trend governed by the constant boron content in the Fe–B–Si substrate. This confirms that the surface coating material does not influence thermal neutron attenuation, which is dominated by ^10^B(n,*α*)^7^Li absorption reactions. Accordingly, neutron performance is presented as efficiency (%) only, consistent with our measurement definition and scope.

To benchmark the results against literature values, the measured mean efficiencies were converted into standard metrics. The linear attenuation coefficient is obtained as

(2) $$\mu =-\frac{\ln T}{x},\;T=1-\frac{\eta }{100},$$

where *x* is the coating thickness in cm and η is the measured efficiency. From μ, the half- and tenth-value layers are

(3) $$HVL=\frac{\ln2}{\mu },\;TVL=\frac{\ln10}{\mu }.$$

For gamma rays, the shielding performance can also be expressed as an equivalent Pb thickness:

(4) $$x_{\mathrm{Pb},\mathrm{eq}}=\frac{\mu }{\mu_{\mathrm{Pb}}}\;x,$$

where $\mu_{\mathrm{Pb}}$ is the linear attenuation coefficient of bulk lead. From NISTIR 5632, the mass attenuation coefficients of Pb are ${\left(\mu /\rho \right)}_{\mathrm{Pb}}=\left\{0.110,\;0.0618,\;0.0561\right\}\;{\mathrm{cm}}^{2}\;g^{-1}$ at 661.7, 1173, and 1332 keV, respectively. Using $\rho_{\mathrm{Pb}}=11.34\;g\;{\mathrm{cm}}^{-3}$, this gives $\mu_{\mathrm{Pb}}=\left\{1.25,\;0.70,\;0.64\right\}\;{\mathrm{cm}}^{-1}$.

These density-independent benchmarks show that W-coated bilayers closely approach Pb-coated systems in effective HVL/TVL and equivalent Pb thickness across 661.7–1332 keV, reinforcing their suitability as Pb-free shielding materials.

## 4. Discussion

### 4.1. Dual-Shielding Mechanisms and Material Design

The dual-layer composite architecture developed in this study was designed to provide integrated shielding against both gamma rays and thermal neutrons by combining a boron-containing amorphous Fe–B–Si matrix and high-Z metal surface coatings. Group A samples, which varied in boron content from 13 at% to 21 at%, enabled the evaluation of compositional tuning effects on radiation attenuation behavior.

Experimental results revealed a clear trade-off between gamma-ray and neutron shielding performance. As the boron content increased, gamma-ray attenuation efficiency slightly decreased (e.g., from 50% to 46% at 661.7 keV), while thermal neutron attenuation significantly improved (from 18% to 29%). This divergence is attributed to the contrasting interaction mechanisms: gamma-ray attenuation is dominated by electron density and atomic number (Z), whereas thermal neutron attenuation is governed by nuclear capture, especially the high thermal cross-section of ^10^B [13,19].

The decrease in Fe content associated with higher B levels likely reduced gamma-ray attenuation due to lower Z and material density. However, the results confirm that boron enrichment in the Fe-based matrix is an effective route to enhancing neutron shielding, with only marginal compromises in gamma attenuation. Such compositional adjustability provides design flexibility to match application-specific radiation profiles.

This concept of material-level trade-off management aligns with recent trends in multifunctional shielding composites, particularly those incorporating boron-containing matrices for dual-mode attenuation [31].

The substrate composition was fixed to isolate the coating effects. This variable-control design allows unambiguous interpretation of how coating material and thickness govern γ and *n* attenuation. Triplicate data reproducibility (<2%) further supports coating uniformity and thickness continuity, minimizing void-induced performance loss. Coating continuity and matrix–coating adhesion reduce voiding pathways that could otherwise degrade neutron moderation and γ absorption.

While direct SEM/EDS microstructural characterization was not performed in the present work, the coating homogeneity and filler dispersion quality can be indirectly verified through the high reproducibility of attenuation measurements (standard deviation < 2%). This statistical uniformity strongly supports a macroscopically uniform particle distribution and consistent coating thickness across all specimens. Similar approaches have been widely adopted to validate composite uniformity in polymer–metal and ceramic–polymer systems, where low statistical variability in bulk electrical or mechanical properties correlates with microstructural homogeneity [32,33,34]. Accordingly, the present interpretation aligns with established composite characterization frameworks, providing sufficient materials-science justification for the observed shielding reproducibility.

### 4.2. Comparison Between Pb and W Coatings

In Groups B and C, where the base composition was held constant (Fe 78 at%, B 13 at%, Si 9 at%), the effect of coating material and thickness was systematically isolated. Results showed that increasing surface coating thickness from 80 to 400 µm led to progressive improvements in gamma-ray shielding, consistent with exponential attenuation principles [8,9].

While Pb shows a slight advantage at lower photon energies, the difference narrows at 1–1.3 MeV, supporting W as a viable Pb-free alternative with advantages in environmental safety and structural robustness.

Our findings corroborate earlier reports that tungsten can serve as an effective substitute for Pb in layered shielding configurations [10,35], especially when combined with neutron-absorbing matrix materials.

### 4.3. Implications for Aerospace and Engineering Applications

The composite system evaluated in this work exhibits key features desirable for radiation shielding in aerospace and advanced engineering contexts: dual-mode protection, weight and thickness tunability, and environmental safety. In high-altitude and extraterrestrial environments, exposure to both gamma and neutron radiation is a major hazard [1,7]. Our dual-layer configuration addresses this challenge by integrating a high-Z surface coating with a boron-rich matrix that effectively captures thermal neutrons.

Compared to conventional materials such as Pb or Al, the Fe–B–Si composites developed here offer a more favorable balance between mass, shielding effectiveness, and environmental compliance. The epoxy-based W coatings in particular present a viable Pb-free alternative with excellent mechanical compatibility for structural integration.

Recent studies also emphasize the importance of tailoring multilayer shielding architectures based on mission-specific threat spectra [36]. The present work contributes to this by demonstrating how coating thickness and matrix composition can be flexibly modulated to optimize performance. In this regard, our material design framework aligns well with current directions in radiation protection material development [31,35].

The measured attenuation coefficients and thickness series provide inputs to a physics-based optimization that correlates $\mu (f_{B},t_{c})$ with boron fraction $f_{B}$ and coating thickness $t_{c}$. These datasets can support future semi-empirical or Monte Carlo modeling to predict optimized γ/*n* attenuation configurations.

Taken together, the Fe–B–Si-based composites with W or Pb coatings constitute a promising platform for scalable, multifunctional shielding applications in aerospace vehicles, satellite systems, defense electronics, and portable protective equipment.

## 5. Conclusions

This study emphasized reproducibility and materials transparency by specifying filler purity, particle size, and coating composition (approximately 60–70 wt% metal). Fe–B–Si-based bilayer composites incorporating W-filled epoxy coatings (with Pb benchmarks) demonstrated dual-mode γ–*n* shielding with consistent attenuation across all thickness levels. W-coated systems achieved up to 87% attenuation at 661.7 keV with 400 μm coatings, closely matching Pb (90%) while avoiding toxicity and environmental concerns. Future work will focus on validating the long-term mechanical integrity of the coatings under extended irradiation and developing refined semi-empirical optimization models linking boron content ($f_{B}$) and coating thickness ($t_{c}$) to shielding performance under mission-relevant spectra.

## Figures and Tables

**Table 1:** Elemental composition of Fe-based composites (Group A).

Sample	Fe (at%)	B (at%)	Si (at%)
#1	78	13	9
#2	76	15	9
#3	74	17	9
#4	72	19	9
#5	70	21	9

**Table 2:** Thermal neutron (0.025 eV) shielding efficiencies of Pb- and W-coated Fe–B–Si composite samples as a function of coating thickness.

Sample	Coating	Thickness (μm)	Neutron Shielding (%)
#6–#10	Pb	80–400	18–29
#11–#15	W	80–400	18–29

## Data Availability

The original contributions presented in this study are included in the article. Further inquiries can be directed to the corresponding author.
